# ALK-Positive Anaplastic Large Cell Lymphoma Associated With Hemophagocytic Lymphohistiocytosis

**DOI:** 10.7759/cureus.41427

**Published:** 2023-07-05

**Authors:** Shing Chao, Zaid I Al-Saheli, Wei Zhao, Shamila Ghosh, Vrushali Dabak

**Affiliations:** 1 Internal Medicine, Henry Ford Health System, Detroit, USA; 2 Hematology and Oncology, Henry Ford Health System, Detroit, USA; 3 Pathology and Laboratory Medicine, Henry Ford Health System, Detroit, USA

**Keywords:** hemophagocytic lymphohistiocytosis (hlh), alk, hlh, alcl, lymphoma

## Abstract

Hemophagocytic lymphohistiocytosis (HLH) has been rarely reported as a complication of anaplastic large cell lymphoma (ALCL), especially in the adult population. We herein present a case of a young woman who presented with multiorgan failure and disseminated intravascular hemolysis and was later found to have ALCL-associated HLH. We also review the current literature on ALCL-associated HLH in adult patients, with their respective treatments and outcomes. We discuss the challenges associated with the diagnosis of lymphoma in the setting of HLH and multiorgan failure. Further, given its high mortality rates, we highlight the importance of promptly identifying and treating the underlying etiology of HLH.

## Introduction

Lymphoid neoplasms are classified based on the cell lineage (i.e., B lymphocytes or T lymphocytes), and within each category, they are further subdivided into precursor versus mature lymphoid neoplasms [1]. Anaplastic large cell lymphoma (ALCL) is a type of mature T-cell lymphoma and presents as one of its four distinct forms: primary systemic anaplastic lymphoma kinase (ALK) positive, primary systemic ALK negative, primary cutaneous, and breast implant-associated ALCL. ALCL usually presents with rapidly progressive lymphadenopathy and B symptoms (i.e., fever, night sweats, and weight loss). The incidence of ALCL in the United States is approximately 1% of non-Hodgkin lymphoma in adults [2]. The majority of the ALCL cases seen in children are associated with ALK gene translocation, which is located on chromosome 2p23.

Although rare in adults, hemophagocytic lymphohistiocytosis (HLH) has been reported as a complication of ALCL in case reports. In HLH, there is a dysregulated hyperactivation of cytotoxic T-cells and macrophages leading to high levels of interferon-gamma and other cytokines release. Furthermore, macrophages phagocytize blood cells such as red blood cells, leukocytes, and platelets. HLH can occur due to genetic aberrancies or secondary to an immunological trigger such as infections, inflammatory disorders, and malignancies. The diagnosis of lymphoma-associated HLH is based on documented histological evidence of lymphoma as well as the presence of at least five of eight HLH-2004 criteria, which includes fever, splenomegaly, bicytopenia, hypertriglyceridemia or hypofibrinogenemia, hemophagocytosis, ferritin above 500 mcg/L, low or absent natural killer cell activity, and soluble CD25 elevation [3].

We herein describe a case of a previously healthy young woman, who presented with multiorgan failure and disseminated intravascular hemolysis and was later found to have ALCL ALK-positive associated HLH. Furthermore, we summarize the current literature on HLH presenting as a complication of ALCL in adult patients. Our case highlights the challenges in diagnosing an underlying lymphoma in the setting of HLH as well as emphasizes the importance of prompt recognition and treatment of HLH and its underlying etiology.

## Case presentation

**Figure 1:**
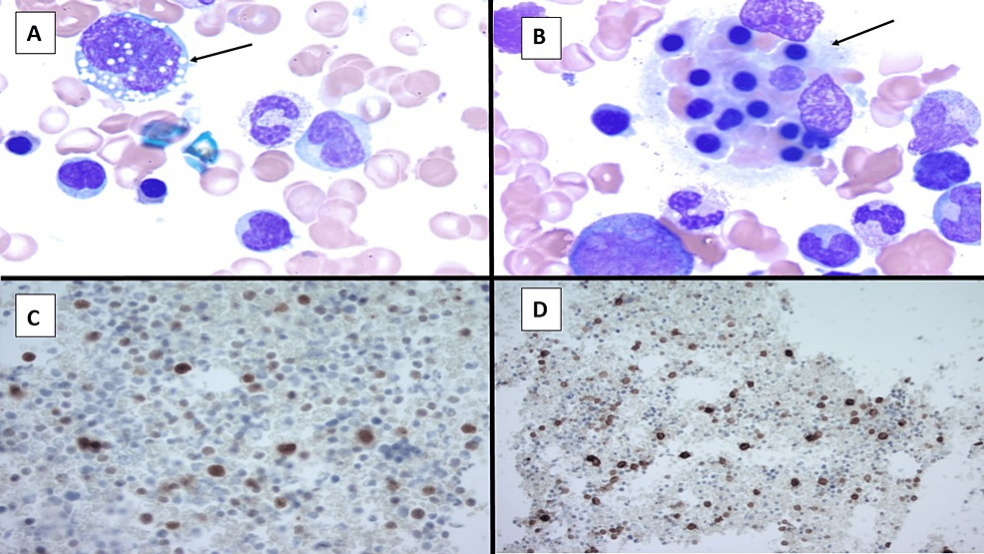
(A-D) ALK-positive ALCL Bone marrow aspirate showing (A) a large atypical lymphoid cell with an irregular nucleus, condensed chromatin, and abundant vacuolated cytoplasm and (B) a hemophagocyte. Immunoreactivity is positive for (C) ALK and (D) CD30.

A previously healthy, 26-year-old woman presented to the emergency department with complaints of fatigue, rhinorrhea, and chest pain. The patient's symptoms were initially thought to be secondary to infectious mononucleosis and managed with supportive care. However, two days later, she had worsening symptoms and developed fever, nausea, dyspnea, and jaundice, requiring hospital admission. She was noted to have tachycardia, tachypnea, hypotension, and hypoxemia. Further workup revealed findings indicative of septic shock with multiorgan failure, including respiratory, hepatic, and renal failure. The patient subsequently developed respiratory distress requiring mechanical ventilation and was started on broad-spectrum antibiotics for possible pneumonia. She continued to quickly deteriorate clinically and had worsening metabolic acidosis requiring emergent dialysis. The respiratory viral panel was negative, including coronavirus and influenza testing. Additional studies were consistent with disseminated intravascular hemolysis but also noted elevated ferritin, elevated triglycerides, low fibrinogen, and bicytopenia (Table 1). Epstein-Barr virus (EBV) deoxyribonucleic acid (DNA) was detected; however, the viral load was below the limit of quantification. Other infectious studies were negative, including but not limited to viral, bacterial, and fungal testing. Given concerns for EBV-related HLH, a bone marrow biopsy was obtained, and the preliminary report showed 15% of atypical lymphoid cells as well as hemophagocytosis (Figure 1). The patient was started on the HLH-94 protocol and received 10 mg/m^2^ of dexamethasone daily and 75 mg/m^2^ of etoposide once. The etoposide dose was reduced by half, given the abnormal liver function studies. After flow cytometry was performed, the atypical lymphoid cells were identified as a clonal T-cell population with aberrant loss of CD3 and CD5 and expression of human leukocyte antigen (HLA)-DR and CD13. Immunohistochemistry was positive for CD30 and ALK (Figure 1). Cytogenetics analysis showed a female chromosome with an aberrant t(2;5)(p23;q35) and fluorescence in situ hybridization identified ALK gene rearrangement. Given these findings, the patient was diagnosed with ALCL ALK-positive. Despite the patient receiving treatment for HLH within two days of admission to the hospital, she continued to quickly deteriorate and had worsening hypoxemia as well as refractory metabolic acidosis and hyperkalemia despite continuous renal replacement therapy. Unfortunately, the patient passed away on day five of hospitalization.

**Table 1:** Diagnostic workup DNA, deoxyribonucleic acid; IgG, immunoglobulin G; IgM, immunoglobulin M; PCR, polymerase chain reaction

Laboratory parameter, (units)	Normal range	On presentation	Day 5
White blood cells, K/uL	3.8-10.6	69.9	32.6
Hemoglobin, g/dL	12.0-15.0	8.0	6.7
Platelets, K/uL	150-450	67	28
Prothrombin time, seconds	11.5-14.5	18.8	94.1
International normalized ratio		1.53	12.48
Partial thromboplastin time, seconds	22-36	42	112
D-dimer, ug/mL	≤0.50	2.47	1.51
Fibrinogen, mg/dL	200-450	175	75
Total lactate dehydrogenase, IU/L	<250	927	11,869
Ferritin, ng/mL	11-307	7,361	12,327
Triglycerides, mg/dL	<200	542	404
Total bilirubin, mg/dL	<1.2	15.9	13.6
Direct bilirubin, mg/dL	0-0.3	9.0	8.2
Alkaline phosphatase, IU/L	40-140	215	747
Aspartate aminotransferase, IU/L	<35	256	7,420
Alanine aminotransferase, IU/L	<52	105	1,015
Creatinine, mg/dL	<1.16	1.70	0.51
Blood urea nitrogen, mg/dL	10-25	38	6
Sodium, mmol/L	135-145	124	135
Chloride, mmol/L	98-111	89	98
Potassium, mmol/L	3.5-5.0	4.7	8.0
Bicarbonate, mmol/L	21-35	17	15
Lactate, mmol/L	<2.1	5.8	23.0
Epstein-Barr virus DNA, quantitative PCR, IU/mL	Undetectable; <50	Detected; <50	
Cytomegalovirus IgM and IgG	Negative	Negative	
Blood and respiratory cultures	No growth	No growth	

## Discussion

Identifying malignancy associated with HLH can be challenging; nevertheless, patients should be evaluated for underlying malignancy if the etiology of HLH is not evident. Relapsed and refractory HLH are also commonly seen in malignancies. In a study by Vick et al., leukemias and lymphomas were associated with 97% of the malignancy associated with HLH, of which T-cell lymphoma was the most prevalent and accounted for 46% of the cases [4]. It is important to note that the management of malignancy associated with HLH includes both the treatment of inflammatory processes associated with HLH as well as treatment of the underlying malignancy. Based on the HLH-94 protocol, induction treatment includes dexamethasone and etoposide followed by a steroid taper [5]. The HLH-94 protocol was developed based on a prospective study that included children aged 15 years or less [5]. As a result, treatment guidelines for HLH in adults are mainly based on studies in pediatric populations. Other treatment options for HLH include allogeneic hematopoietic stem cell transplantation, especially in refractory cases.

A literature review of ALCL-associated HLH in adults is presented in Table 2. Of the 14 cases found in the literature, eight patients improved and six patients died [6-18]. Seven patients were ALK-positive, five ALK-negative, and two ALK unknown. Most of the patients were noted to have negative infectious workup. The treatment approach for HLH varied but most patients received a combination of steroids with etoposide or intravenous immunoglobulins. Malignancy associated with HLH has a high mortality rate, hence early diagnosis and treatment lead to better outcomes. However, the underlying malignancy is not always obvious, just like the patient presented in this case report, and many patients, unfortunately, die before effective malignancy treatment can be provided.

**Table 2:** Review of literature on ALCL-associated HLH in adults (age above 18 years) TIA-1, T-cell intracellular antigen-1; EBV, Epstein-Barr virus; LCA, leukocyte common antigen; EMA, epithelial membrane antigen; Ig, immunoglobulin; ALK, anaplastic lymphoma kinase

Authors (year)	Study type	Age, years	Diagnosis	Markers	HLH present	Positive infectious workup	HLH treatment	Outcome
Shimizu et al (2010) [6]	Case report	39	Primary cutaneous anaplastic large-cell lymphoma	Positive for CD2, CD8, CD25, CD30, and granzyme B. Negative for ALK, CD3, CD4, CD5, CD20, CD56, and TIA-1.	Yes	Negative	Radiation of cutaneous lesion	Improved
Machaczka et al (2011) [7]	Case report	22	ALK-positive anaplastic large T- cell lymphoma	Positive for ALK	Yes	Negative	IV Ig and corticosteroids	Improved
Basheer et al (2014) [8]	Case report	56	Cutaneous anaplastic large cell lymphoma	Positive for LCA, CD3, and CD30. Negative for pan-cytokeratin, CD20, ALK, and EMA.	Yes	Unknown	Steroids	Died
Mayson et al (2014) [9]	Case report	64	ALK-negative anaplastic large-cell lymphoma	Negative for ALK	Yes	Positive EBV PCR	Dexamethasone, etoposide, and rituximab	Improved
Xu and Burns (2014) [10]	Case report	52	ALK-negative anaplastic large-cell lymphoma	Positive for CD3, CD4, CD25, CD30, EMA, and p53. Negative for ALK	Yes	HIV positive	Unknown	Died
Akavia and Krause (2017) [11]	Case report	22	Anaplastic large cell lymphoma	Unknown	Yes	Unknown	HLH-94 treatment protocol	Died
Ibrahim et al (2018) [12]	Case report	69	ALK-positive anaplastic large-cell lymphoma	Positive ALK	Yes	Negative	Dexamethasone and etoposide	Improved
Pasvolsky et al (2019) [13]	Case Series	Patient 1: 68; Patient 2: 64	Patient 1: ALK-negative anaplastic large cell lymphoma; Patient 2: anaplastic large cell lymphoma	Patient 1: ALK-positive; Patient 2: unknown	Patient 1: yes; Patient 2: yes	Patient 1: unknown; Patient 2: unknown	Patient 1: dexamethasone and etoposide; Patient 2: IV Ig and dexamethasone	Patient 1: Died; Patient 2: Improved
Angelova et al (2020) [14]	Case report	50s	ALK-positive anaplastic large-cell lymphoma	Positive for CD30, ALK, perforin, EMA, CD4, and CD3	Yes	Positive anti-EBV IgG	Prednisone	Died
Ud Din et al (2020) [15]	Case report	44	ALK-positive anaplastic large T-cell lymphoma	Positive for CD30 and ALK	Yes	High IgG titers for Bartonella henselae and Coxiella burnetii	Dexamethasone and etoposide	Improved
Tariq et al (2022) [16]	Case report	55	ALK-positive anaplastic large-cell lymphoma	Positive for ALK	Yes	Negative	Steroids and etoposide	Improved
Nogueira et al (2023) [17]	Case report	25	ALK-positive anaplastic large-cell lymphoma	Positive for CD30 and ALK	Yes	Negative	Treatment of underlying malignancy with cyclophosphamide, doxorubicin, vincristine, etoposide, and prednisolone	Improved
Wentzell S et al (2023) [18]	Case report	39	ALK-negative anaplastic large-cell lymphoma	Positive for CD3, CD30, and granzyme. Negative for ALK.	Yes	Unknown	Unknown	Died

## Conclusions

Lymphoma associated with HLH in adults, although rare, is associated with high mortality rates secondary to multiorgan failure. This patient’s initial presentation of septic shock and disseminated intravascular hemolysis created a confounding factor given several overlapping clinical features between these pathologies. Therefore, it is important to promptly recognize the signs and symptoms of HLH as well as its underlying etiology.
